# Prevalence, correlates and patterns of waterpipe smoking among secondary school students in southeast London: a cross-sectional study

**DOI:** 10.1186/s12889-016-2770-1

**Published:** 2016-02-01

**Authors:** Mohammed Jawad, Gerald Power

**Affiliations:** 1 Department of Primary Care and Public Health, Imperial College London, Hammersmith, W6 8RP UK; 2 Academic Unit of Primary Care and Population Sciences, University of Southampton, Southampton, Hampshire SO16 6YD UK; 3 Community and Safety Enforcement on behalf of the South East London Illegal Tobacco Network, Southwark Council, London, SE1 2QH UK

**Keywords:** Waterpipe smoking, Hookah, Shisha, Young people, Tobacco

## Abstract

**Background:**

Waterpipe smoking is growing worldwide, but little is known of its epidemiology in the UK due to its absence from national health surveys. We sought to address this by calculating the prevalence of waterpipe smoking among secondary school students in southeast London.

**Methods:**

We conducted a pooled secondary analysis of routine health surveillance surveys among 11–17 year olds in convenience-sampled secondary schools from three ethnically-diverse areas of southeast London. We calculated ever (lifetime) waterpipe use, and compared its sociodemographic correlates to ever (lifetime) cigarette use. In one area we collected data on patterns of waterpipe use.

**Results:**

Of 2,098 respondents (mean age 14.1 ± 1.7 years, 55.7 % male, 46.6 % of black ethnicity), ever waterpipe use was 39.6 % (95 % CI 37.6–41.7 %) and was higher than that for ever cigarette use (32.4 %; 95 % CI 30.5–34.4). While waterpipe users were significantly and independently more likely to be male and of non-white ethnicities, at least 30 % of all age, gender and ethnic sub-groups had tried waterpipe smoking. In contrast, cigarette users were more likely to be older and of white ethnicity. In one of the three areas, over a quarter of waterpipe users were occasional or regular waterpipe smokers, and most were introduced to and currently used waterpipe in waterpipe-serving premises or friends’ homes.

**Conclusions:**

Waterpipe smoking prevalence was high in southeast London, and users exhibited a different sociodemographic profile to cigarette users. Waterpipe should be included in national health surveys of young people. National surveillance is warranted to help develop suitable interventions to prevent uptake and promote cessation.

## Background

Waterpipe smoking, a nicotine delivery device where tobacco smoke is passed through water prior to inhalation, is endemic in the Middle East and Indian subcontinent, but is growing in prevalence worldwide [1–3]. Data among adults from Europe suggest up to 20 countries now have waterpipe as its second most prevalent tobacco type after cigarettes, and the highest current rates found in Latvia (11.5 %), Lithuania (9.0 %), Cyprus (8.5 %) and Denmark (8.4 %) [4]. Among young people, past-30 day waterpipe prevalence is highest in Lebanon (36.9 %), West Bank (32.7 %) and parts of Eastern Europe (Latvia 22.7 %, Czech Republic 22.1 %, Estonia 21.9 %) [5].

Central to the growth in waterpipe smoking is the perception that it is a safer mode of tobacco consumption than cigarettes. A recent systematic review of attitudes to waterpipe smoking affirmed this and added that the main motives for use were socialising, relaxation, pleasure and entertainment [6]. Users are drawn to the array of flavours that are added to waterpipe tobacco, and the pipe is generally shared with peers over a 30 to 60 min period [7, 8].

Yet the evidence suggests that waterpipe smoking is perhaps as harmful as cigarette smoking. A recent meta-analysis on the health effects of waterpipe smoking concluded that it was significantly associated with lung cancer, low birth weight, periodontal disease and respiratory diseases in children [9]. Further studies show a link between waterpipe smoking and heart disease [10, 11] and chronic obstructive pulmonary disease [12–14]. Regular users are known to exhibit signs of nicotine dependence such as cravings and withdrawal symptoms [15–17], and a review of the literature suggested that daily waterpipe smokers absorb as much nicotine as a 10-a-day cigarette smoker [18].

Further research is therefore warranted on this emerging public health concern. In the United Kingdom (UK), the British Heart Foundation calculated that the number of waterpipe-serving premises had grown 210 % between 2007 and 2012, and recent estimates suggest 400 waterpipe-serving premises can be found in London alone [19]. However waterpipe tobacco smoking remains absent from national health surveys, so it is unknown to what extent this practice is spreading and to whom. Little is known of its epidemiology, especially among young people, which is concerning considering anecdotal reports of regular underage sales. This study therefore aimed to evaluate the prevalence, correlates and patterns of waterpipe users among young people in a diverse ethnic urban area of the UK.

## Methods

### Design, setting and sampling

We analysed secondary data from cross-sectional surveys of secondary school students in three inner southeast, ethically-diverse London Boroughs (districts): the Royal Borough of Greenwich, the London Borough of Lambeth, and the London Borough of Southwark [20].

The sample frame was all Year 8 (typically aged 12-13 years) and Year 10 (typically aged 14-15 years) students registered in schools enlisted with the local education authorities of each borough. All schools were invited to participate in routine surveillance surveys conducted by local governments. Within each school all Year 8 and Year 10 students were asked to be sampled, and parental assent was gained. Students completed a self-administered questionnaire between April and June 2014, with schools having the option of using paper or online surveys. A response rate was not determined by any local government surveillance group. As this study was a secondary analysis of routinely collected, anonymised data, no ethics approval was required.

### Questionnaire and measures

For the Greenwich sample a 23-item questionnaire was distributed covering patterns of tobacco use and socio-demographic information. Waterpipe tobacco questions included prevalence, frequency, age of initiation, the setting of initial and continued use, the cost of use, and family and friend waterpipe use. Socio-demographic data included gender, age, and ethnicity.

For both the Lambeth and Southwark samples a 97-item questionnaire called the Health-Related Behaviour Questionnaire was distributed covering food and diet, substance use including tobacco, health and safety, relationships and mental health, leisure and money, exercise, and socio-demographic information. A single waterpipe tobacco question was used to derive prevalence data.

We used two binary outcome measures: ever (lifetime) cigarette smoking and ever (lifetime) waterpipe smoking. For the Greenwich sample, this was defined as answering ‘Yes’ to the questions “Have you ever smoked a cigarette/shisha pipe before?” (Yes/No). For the Lambeth and Southwark samples, this was defined as answering anything other than ‘Never’ to the questions “Which statement describes you best?” (I have never smoked at all, not even a puff/I have tried smoking once or twice/I used to smoke, but I don’t know/I smoke occasionally (less than 1 cigarette a week)/I smoke regularly but would like to give it up/I smoke regularly and don’t want to give it up) and “Have you ever used a shisha?” (Never/In the last month/In the three months/In the last 6 months/In the last twelve months/More than twelve months ago). Independent variables included age (11–14/15–17), gender, and ethnicity (white/black/other) and concurrent ever cigarette or ever waterpipe use. Of 2,575 initial responses, we removed observations with missing data in outcome measures (cigarette use, *n* = 81; waterpipe use, *n* = 39) or independent variables (age, *n* = 117; sex, *n* = 34; ethnicity, *n* = 62), and excluded those aged 18 or over (*n* = 141, all from Greenwich survey), resulting in 2,098 observations available for analysis (81.5 % of the original responses).

### Statistical analysis

We analysed data descriptively using counts and percentages for categorical data, means and standard deviations for normally-distributed continuous data, and medians and interquartile ranges for skewed continuous data. We calculated the prevalence of waterpipe and cigarette smoking by independent variables. We conducted forced multivariate logistic regression models to test the association between independent variables and our two outcome measures, ever cigarette and ever waterpipe smoking. We used a two-level, mixed effects design with random intercept to account for the effect of clustering in the three different boroughs (Stata command: xtmelogit), but did not have all necessary data to cluster at school-level. We tested for multi-collinearity between independent variables using the variance inflation factor, the mean of which was 1.15 for both models, enabling us to assume reasonable independence between variables. We used a significance level of 5 % in all analyses. All analyses were conducted in Stata 12.0 (StataCorp).

## Results

### Characteristics of sample

Eight schools were from Lambeth (school response rate 47.1 %), and six schools were from Southwark (school response rate 33.3 %). We were not provided with school IDs for the Greenwich sample. Table 1 presents the characteristics of our sample, including 95 % confidence intervals. Of 2,098 respondents, the majority were either from Lambeth (41.2 %, *n* = 865) or Southwark (40.7 %, *n* = 854). The mean age was 13.7 ± 1.3 years and 55.1 % (*n* = 1157) were male. Most considered themselves of black (47.3 %, *n* = 993) or white (30.4 %, n = 637) ethnicity. Based on the sampling frame we expected the age range to be 10–15 years, however on breaking down the sample by borough it appeared that the Greenwich survey recruited 16–18 year old students (Years 11-12). We therefore compared the characteristics of our sample to 2011 census data from the same area [21] (Table 1), which showed the sample had an under-representation of females, those of white ethnicity, and those from Greenwich (the latter due to exclusion of responses by adults).

**Table 1:** Characteristics of sample (*N* = 2,098)

Characteristic	Our sample	Reference sample
Age, mean ± SD	*13.7 ± 1.3*	*-*
Age, % (95 % CI)		
- 11–14 years	*52.6 (50.5–54.8)*	*35,103 (57.2)*
- 15–17 years	*47.4 (45.2–49.5)*	*26,289 (42.8)*
Borough, *n* (%)		
- Lambeth	*41.2 (39.1–43.3)*	*21,119 (34.4)*
- Southwark	*40.7 (38.6–42.8)*	*20,345 (33.1)*
- Greenwich	*18.1 (16.4–19.7)*	*19,928 (32.4)*
Gender, *n* (%)		
- Female	*44.9 (42.7–47.0)*	*128,333 (50.4)*
- Male	*55.1 (53.0–57.2)*	*126,224 (49.6)*
Ethnicity, *n* (%)		
- White	*30.4 (28.4–32.3)*	*488,376 (57.7)*
- Black	*47.3 (45.2–49.5)*	*204,708 (24.2)*
- Other	*22.3 (20.5–24.1)*	*152,842 (18.1)*

Note: Reference sample taken from 2011 local census data: age data pooled for all three boroughs; gender and ethnicity data are from all residents pooled for all three boroughs; borough data are age-specific to our sample

### Prevalence and correlates of cigarette and waterpipe use

Table 2 presents the prevalence and correlates of cigarette and waterpipe use. The prevalence of ever cigarette use was 31.8 % (95 % CI 29.8–33.8). Compared to those aged 11–14 years, cigarette use was significantly higher among those aged 14–17 years (AOR 2.23, 95 % CI 1.71–2.89). There were no gender differences in cigarette use. Compared to those of white ethnicity, cigarette use was significantly lower among those of black ethnicity (AOR 0.48, 95 % CI 0.35–0.65) and other ethnicity (AOR 0.50, 95 % CI 0.35–0.65). Compared to never waterpipe users, cigarette use was significantly higher among ever waterpipe users (AOR 42.33, 95 % CI 30.75–58.26).

**Table 2:** Prevalence and correlates of cigarette and waterpipe smoking

Characteristic	Cigarettes		Waterpipe	
	*n* (%)	AOR (95 % CI)	*n* (%)	AOR (95 % CI)
Overall	*667 (31.8)*		*841 (40.1)*	
Age				
- 11–14 years	*286 (25.9)*	*1.00*	*387 (35.1)*	*1.00*
- 14–17 years	*381 (38.3)*	2.23 (1.71, 2.89)**	*454 (45.7)*	*1.07 (0.84, 1.35)*
Gender				
- Female	*295 (31.4)*	*1.00*	*327 (34.8)*	*1.00*
- Male	*372 (32.2)*	*0.82 (0.63, 1.07)*	*514 (44.4)*	1.47 (1.16, 1.87)*
Ethnicity				
- White	*198 (31.1)*	*1.00*	*201 (31.6)*	*1.00*
- Black	*316 (31.8)*	0.48 (0.35, 0.65)**	*440 (44.3)*	2.40 (1.79, 3.20)**
- Other	*153 (32.7)*	0.50 (0.35, 0.72)**	*200 (42.7)*	2.15 (1.54, 3.02)**
Cigarette smoker				
- No	*-*	*-*	*283 (19.8)*	*1.00*
- Yes	*-*	*-*	*558 (83.7)*	42.04 (30.60, 57.77)**
Waterpipe smoker				
- No	*109 (8.7)*	*1.00*	*-*	*-*
- Yes	*558 (66.4)*	42.33 (30.75, 58.26)*	*-*	*-*

*p < 0.01, **p < 0.001; two-level random intercept model which accounts for clustering at borough-level

The prevalence of ever waterpipe use was 40.1 % (95 % CI 38.0–42.2). There were no age differences in waterpipe use. Compared to females, waterpipe use was significantly higher among males (AOR 1.47, 95 % CI 1.16–1.87). Compared to those of white ethnicity, waterpipe use was significantly higher among those of black ethnicity (AOR 2.40, 95 % CI 1.79–3.20) and other ethnicity (AOR 2.15, 95 % CI 1.54–3.02). Compared to never cigarette users, waterpipe use was significantly higher among ever cigarette users (AOR 42.04, 95 % CI 30.60–57.77).

### Patterns of waterpipe tobacco use (Greenwich-only survey)

Of 74 (out of 84) ever waterpipe users that answered questions on frequency of use, the majority (62.2 %, *n* = 46) had only smoked once or twice, 12.2 % (*n* = 9) were former waterpipe users, 16.2 % (*n* = 12) smoked occasionally but not every week, and 9.5 % (*n* = 7) smoked regularly. Nobody in the sample smoked waterpipe on a daily basis. Using the above data we calculated the prevalence of current (occasional or regular) waterpipe smoking as 5.0 % (*n* = 19) in Greenwich.

Most users usually shared only one waterpipe (80.5 %, *n* = 70), and the remainder shared several (19.5 %, *n* = 17). Most users smoked at the weekend (73.6 %, *n* = 64) compared to weekdays (26.4 %, *n* = 23). Only one respondent owned a waterpipe.

### Setting of initiation and current use (Greenwich-only survey)

The mean age of waterpipe initiation was 14.2 ± 1.6 years. Nearly three quarters (73.6 %, *n* = 39) had family or friends that used waterpipe. The most common setting where waterpipe was initiated was at a friend’s house (32.5 %, *n* = 40), or at waterpipe-serving premises (31.7 %, *n* = 39). Others initiated waterpipe at home (16.3 %, *n* = 20) or elsewhere (19.5 %, *n* = 24). Waterpipe was first introduced to users by a friend or person their age (44.4 %, *n* = 32), by buying it themselves (22.2 %, *n* = 16), by an adult they knew (18.1 %, *n* = 13), by a relative (9.7 %, *n* = 7) or by someone their age they didn’t really know (5.6 %, *n* = 4).

Continued waterpipe use also commonly occurred at waterpipe-serving premises (38.0 %, *n* = 30), at a friend’s house (27.9 %, *n* = 22). Other continue to use waterpipe at home (15.2 %, *n* = 12) or elsewhere (18.9 %, *n* = 15). Continued waterpipe use was commonly with friends or family at someone else’s home (33.3 %, *n* = 18), with a group of friends at a café or bar (29.6 %, *n* = 16), with friends or family at home (18.5 %, *n* = 10), or with someone else (18.5 %, *n* = 10). The median cost reportedly paid for a single waterpipe in a waterpipe-serving premise was £10 (IQR £6–15, range £5–30).

## Discussion

### Main results

This study demonstrates that two-fifths of 2,231 secondary school students mainly aged 12–15 years in southeast London had tried waterpipe smoking. This is higher than the third of students who had tried cigarettes. We found important sociodemographic differences in tobacco use: while cigarette use was associated with older white students, waterpipe use is associated with non-white males. Despite this at least 30 % of all age, gender and ethnic sub-groups had tried waterpipe smoking.

Our findings shed light on important patterns of use. Despite the high level of waterpipe experimentation, only a quarter of ever waterpipe users were occasional or regular users and nobody used it daily. This may reflect limits to access, high cost for single users, and its dependence on peers/family to also be available to smoke. However, this finding may also be a reflection of the sampling process, and with small numbers it is difficult to firmly explain the absence of daily users. A worrying proportion (26 %) of ever waterpipe users were introduced to it by a relative or adult, which is concerning and probably emphasises the level of social acceptability towards this product.

### Previous research

There is an absence of nationally-representative data on waterpipe smoking among young people in the UK. Our measured waterpipe prevalence of ever use (40.1 %) is one and a half times higher than among a predominantly south Asian sample from northwest London (24.0 %) [22], and over three times higher than among a predominantly white sample from a small northern English city (12.0 %) [23]. This study’s lifetime prevalence is also higher than that measured in other settings such as Canada (10.1 %) [24], the US (7.3 %) [25], Jordan (25.9 %) [26], and Oman (26.6 %) [27], although Lebanon remains the country with the highest lifetime prevalence among young people (44.3 %) [28].

In our study cigarette use was strongly associated with waterpipe smoking, and vice versa, and several studies have explored this relationship further. Longitudinal reports among young people in the US and Denmark have shown that waterpipe is significantly associated with initiation of cigarettes [29], and higher intensity of cigarette use [30]. One randomised controlled cessation trial among cigarette smokers in Syria found that several participants initiated waterpipe smoking while attempting to quit cigarettes [31]. These studies underscore the importance of tobacco’s addictive properties by whichever route and are tentative suggestions of waterpipe’s potential as a gateway product to cigarettes and undermine the progress made in tobacco control [32].

### Research and policy implications

Our study identifies several research and policy implications. Considering the sociodemographic profile of waterpipe smokers is different to that of cigarette smokers, waterpipe-specific interventions should be developed to prevent uptake and promote cessation. The strong association between non-white ethnicities and waterpipe smoking suggests these interventions should involve addressing cultural aspects of waterpipe use. One example is attending to the fact that population groups may use waterpipe-serving premises as a socially-acceptable alternative to alcohol consumption [33], or as an expression of cultural heritage [34]. Having said this, nearly a third of white students had tried waterpipe smoking, emphasising the cross-cultural nature of this habit.

We identified that most students in this study initiated waterpipe under 18 years of age, the legal age of tobacco sale in the UK. Considering a third of users initiated waterpipe in waterpipe-serving premises (and indeed 38 % continue to use waterpipe there), proximity to such premises may promote uptake [22, 35]. While taxation is thought to be among the single most effective tobacco control intervention to reduce cigarette demand [36, 37], waterpipe tobacco may be more inelastic than cigarettes as consumers generally share waterpipes, and hence its cost, with their peers [38]. Waterpipes consumed by one person may be more price senstive than waterpipes consumed by group sharing. Efforts should be made to enforce health warning labels on waterpipe tobacco packs, apparatuses and related accessories in line with recommendations of the WHO Framework Convention on Tobacco Control, as this appears poorly implemented in some settings [39]. In London particularly, these recommendations may prove difficult to implement without further financial support of tobacco control enforcement work.

### Strengths and limitations

This study benefits from its large and ethnically-diverse sample size, covering three areas of London. Although our sample demographics were not completely consistent with local census data, our findings may be generalizable to other ethnically-diverse urban areas of the country. However this study has several limitations. We were unable to include measures of socioeconomic position in our multivariate analysis, although a recent epidemiological review suggested waterpipe smoking is generally associated with a higher socioeconomic status [2, 22]. We were unable to describe other measures of waterpipe prevalence, such as current or frequent use (due to small sample sizes), which may better inform tobacco control surveillance. Our sample may have been prone to selection bias as we are unsure of the characteristics of non-respondents, both at school-level and student-level.

## Conclusions

Ever waterpipe smoking is common in young school age children, and more prevalent than cigarettes, in an ethnically-diverse area of southeast London. Correlates of waterpipe use may vary considerably to those for cigarette use. National tobacco control surveillance should include gathering waterpipe prevalence data to inform epidemiological trends in use. Meanwhile, appropriate interventions should be designed and evaluated, and policy should be enforced, to prevent further uptake and promote cessation.
